# Comprehensive Review on Medicinal Applications of Coumarin-Derived Imine–Metal Complexes

**DOI:** 10.3390/molecules27165220

**Published:** 2022-08-16

**Authors:** Siddappa A. Patil, Vishal Kandathil, Anjali Sobha, Sasidhar B. Somappa, Max R. Feldman, Alejandro Bugarin, Shivaputra A. Patil

**Affiliations:** 1 Centre for Nano and Material Sciences, Jain University, Jain Global Campus, Bangalore 562112, India; 2 Organic Chemistry Section, Chemical Sciences & Technology Division, National Institute for Interdisciplinary Science and Technology (CSIR), Thiruvananthapuram 695019, India; 3 Department of Chemistry & Physics, Florida Gulf Coast University, 10501 FGCU Boulevard South, Fort Myers, FL 33965, USA; 4 Pharmaceutical Sciences Department, College of Pharmacy, Rosalind Franklin University of Medicine and Science, 3333 Green Bay Road, North Chicago, IL 60064, USA

**Keywords:** coumarin-derived imine–metal complexes, antimicrobial, anticancer, antioxidant, anthelmintic, pesticidal

## Abstract

**Simple Summary:**

This review is an attempt to gather, analyze, and systemize data about the antimicrobial, anticancer, antioxidant, anthelmintic, pesticidal, and nematocidal properties of coumarin-derived imine–metal complexes. With these properties is a discussion of the progress medicinal chemistry has made on these complexes, and the findings presented here show a promising future for this field. We hope that our review will provide medicinal chemists and biologists with a thorough and accurate review of coumarin-derived imine–metal complexes and encourage them to identify investigational new drug (IND) candidates. With those IND candidates there is the hope of biologically active drugs and a healthier, happier future for humanity.

**Abstract:**

Coumarins are fused six-membered oxygen-containing benzoheterocycles that join two synthetically useful rings: α-pyrone and benzene. A survey of the literature shows that coumarins and their metal complexes have received great interest from synthetic chemists, medicinal scientists, and pharmacists due to their wide spectrum of biological applications. For instance, coumarin and its derivatives have been used as precursors to prepare a large variety of medicinal agents. Likewise, coumarin-derived imine–metal complexes have been found to display a variety of therapeutic applications, such as antibacterial, antifungal, anticancer, antioxidant, anthelmintic, pesticidal, and nematocidal activities. This review highlights the current synthetic methodologies and known bioactivities of coumarin-derived imine–metal complexes that make this molecule a more attractive scaffold for the discovery of newer drugs.

## 1. Introduction

**Figure 1:**
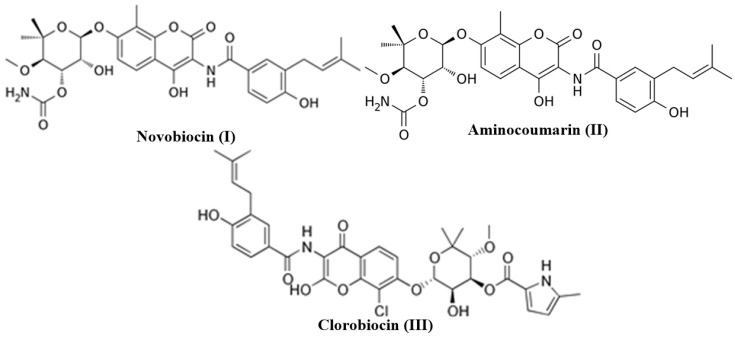
Clinically used coumarin-based antibiotic drugs.

The oxygen-containing benzo-fused heterocycles, such as coumarins (2H-chromen-2-ones), are one of the most important and considered classes of compounds in medicinal chemistry because of their widespread pharmacological activities [1]. The simplest member of the coumarin family of compounds, “coumarin”, was isolated by Vogel in the year 1920 and eventually prepared by Sir William Henry Perkin through the Perkin reaction in 1868 [2,3]. Over the last few decades, researchers have explored the coumarin derivatives for various important biological activities such as anti-inflammatory, antioxidant, antithrombotic, antiallergic, antiviral, and anticancer [4,5,6,7,8,9,10,11]. Medicinal chemists continue to discover novel coumarins of natural (plant extracts) and unnatural (synthetic) analogs to further improve the currently identified biological activities while uncovering new medicinal uses. Coumarins have been considered as ideal small molecule candidates for the drug discovery and development process because they possess drug-like properties such as high solubility, low molecular weight, high bioavailability, and low toxicity along with their diverse biological activities [12,13]. Some notable coumarin analogs such as novobiocin (**I**), aminocoumarin (**II**), and clorobiocin (**III**) have been clinically used as antibiotic drugs [14,15,16] (Figure 1). At present, coumarin pharmacophore has been considered a privileged scaffold because of its medicinal aspects.

**Figure 2:**
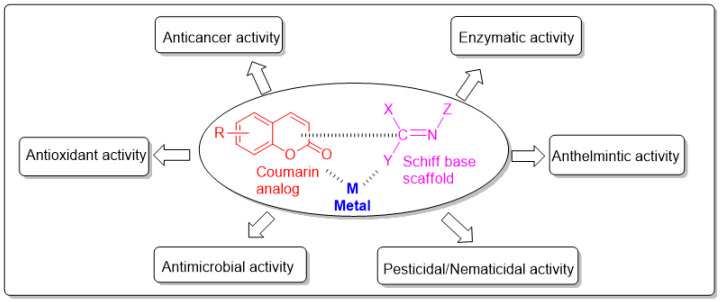
Important biological activities of the coumarin-derived imine–metal complexes.

In recent years, Schiff bases have been used as multipurpose scaffolds to obtain biologically important molecules. Particularly, Schiff bases complexed with metal will have further enhanced biological activity. Therefore, the combination of Schiff base metal complexes with pharmacologically important small-molecule organic ligands such as coumarins is one of the important medicinal chemistry strategies to obtain ideal drug candidates. [17] (Figure 2). 

**Figure 3:**
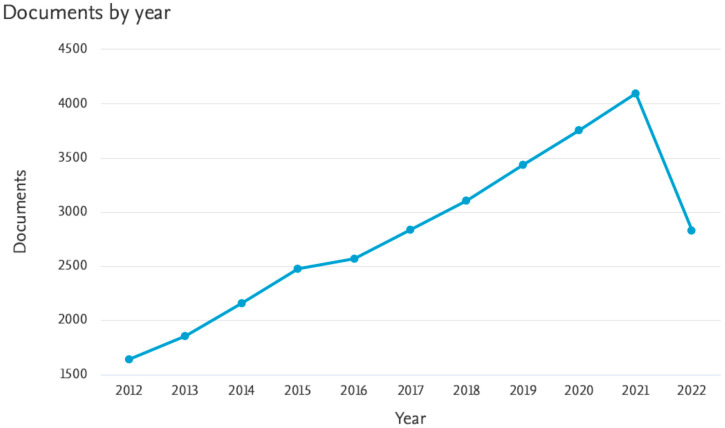
Articles published on coumarin–metal complexes from 2012–2022.

Coumarin pharmacophores are a part of several active drugs, such as the vitamin K antagonist, Warfarin. Because it can be assumed that coumarin–metal complexes will have important medical applications, we have provided a chart (Figure 3) that includes the articles published on these complexes indexed in Scopus for the past ten years (2012–2022). We recently reviewed medicinal applications of coumarins bearing azetidinone and thiazolidinone moieties [18]. In continuation of our effort on coumarin chemistry, we have mainly focused on its Schiff base–metal complexes as possible pharmacological agents in this review. 

## 2. Chemistry

Coumarins are therapeutically active members of the benzopyran-2-one family. Coumarins are extensively dispersed in nature and can be found in both naturally occurring and synthetic medicinally active compounds. In recent years there has been considerable growth in the chemistry of coumarins as a keystone for the design and development of a considerable number of compounds [19,20]. Nowadays, coumarin and its derivatives, especially Schiff bases derived from coumarins, belong to the most active classes of compounds and possess a wide spectrum of biological activity [21,22]. On the other hand, metal complexes derived from Schiff base ligands of coumarin show tremendous potential in numerous fields such as fluorescent probes, optical brighteners, antioxidants, antimicrobials, anthelmintics, hypotensive, and inhibitors of platelet aggregation and cytotoxic activity [23,24,25,26,27,28,29,30]. Hence, the synthesis, structural identification, and biological activity evaluation of new derivatives of coumarin-derived imine–metal complexes continually pique research interest across the world.

## 3. Medicinal Applications of Coumarin-Derived Imine–Metal Complexes

### 3.1. Antimicrobial Activity of Coumarin-Derived Imine–Metal Complexes

**Figure 4:**
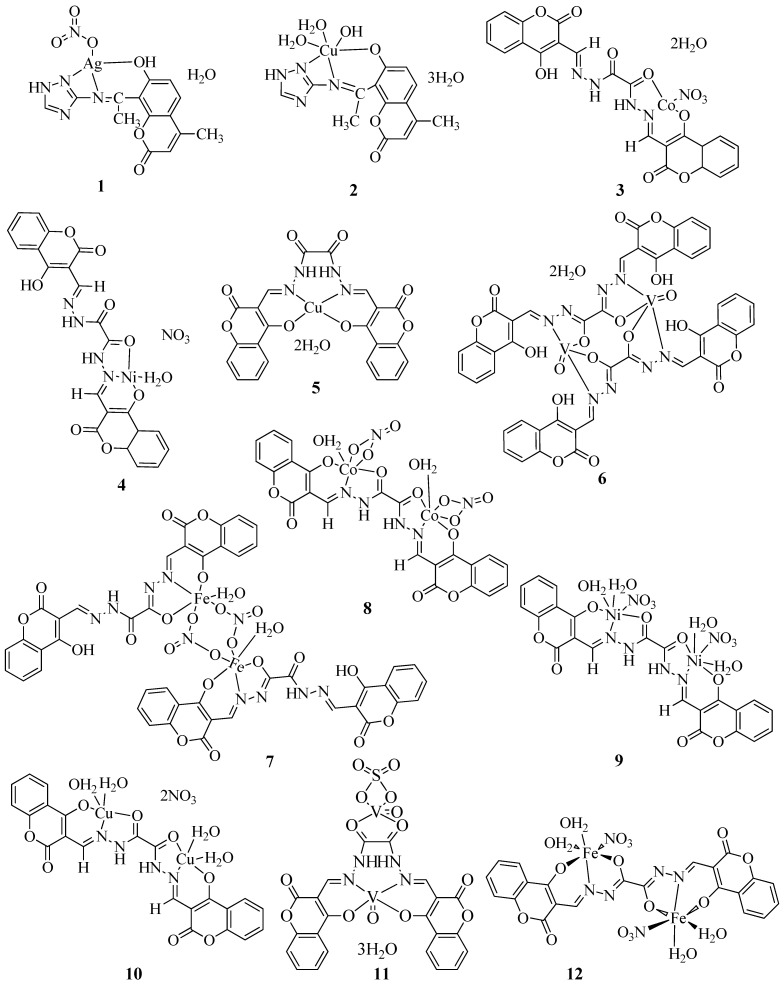

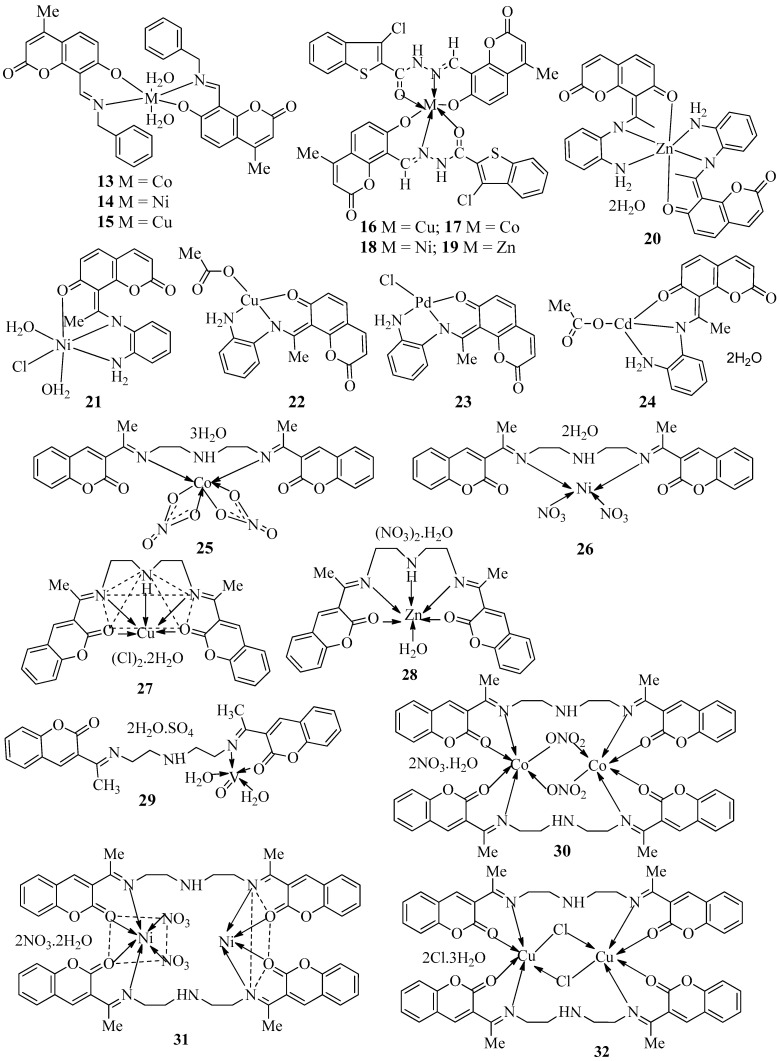

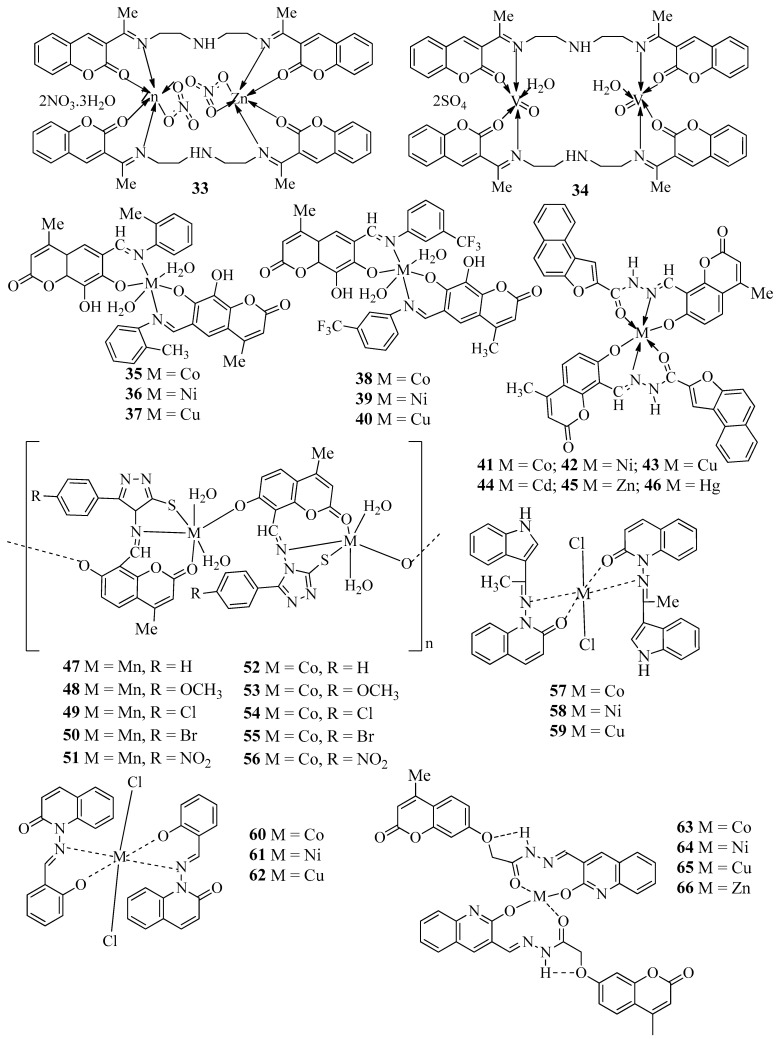

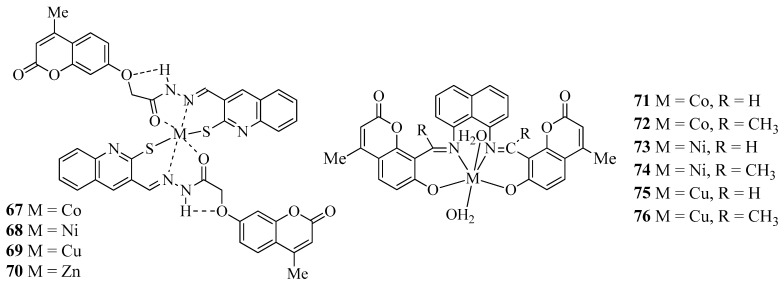
Structures of coumarin-derived imine–metal complexes (**1**–**76**).

Farghaly et al. synthesized a Schiff base (Sbat) using 8-acetyl-7-hydroxy-4-methyl coumarin and 3-amion-1,2,4-triazole [31]. The reaction of the Schiff base with Ag(I) and Cu(II) metal ions produced the [Ag(Sbat)(NO_3_)]H_2_O (**1**) and [Cu(Sbat)(OH)(H_2_O)_2_].3H_2_O (**2**) complexes (Figure 4). The antimicrobial properties of Gram-positive bacteria *Staphylococcus aureus* (*S. aureus*), *Staphylococcus faecalis* (*S. faecalis*), and *Bacillus subtilis* (*B. subtilis*) were evaluated using the standard agar diffusion test and Gram-negative bacteria used included *Escherichia coli* (*E. coli*), *Neisseria gonorrhoeae* (*N. gonorrhoeae*), and *Pseudomonas aeruginosa* (*P. aeruginosa*) (Table 1). Antifungal activity against *Aspergillus flavus* (*A. flavus*) and *Candida albicans* (*C. albicans*) were also studied with the prepared complexes (Table 1). From the activity studies obtained, it was understood that the Schiff base had no activity against the two fungus species. Compounds **1** and **2** show no inhibitory action against the fungus. Sbat was found to be active against *E. coli*, *N. gonorrhoeae*, and *S. faecalis*. Also, **1** exhibited better antibacterial activity than the Schiff base. Complex **2** did not show any antibacterial activity against *S. faecalis* and *N. gonorrhoeae*. Against all microorganisms, **1** showed the maximum activity. The fact that the silver(I) complex was identified in a nanostructure might explain its biological activity because small silver nanoparticles with large surface area interact with bacteria better than larger ones. Also, it is worth noting that the reduced lipophilicity of **2** explains why it has weaker antibacterial activity than its parent ligands.

By condensing 3-formyl-4-hydroxycoumarin and oxalyldihydrazide in a 2:1 molar ratio, Linert et al. produced a hydrazone Schiff base ligand. In molar ratios of 1:1 or 2:1 (M:L), the Schiff base ligand interacted as a mono-, bi-, tri-, or even tetradentate ligand with metal cations to form mono- or binuclear complexes as keto or enol isomers, where M = Co(II), Ni(II), Cu(II), VO(IV), and Fe(III) (**3**–**12**) (Figure 4) [32]. Both the Schiff base ligand and its metal complexes were tested against Gram-positive and Gram-negative bacteria along with one type of fungi. Against these microorganisms, the synthesized complexes showed modest antibacterial and antifungal activity (Table 1). Chelation tends to make the ligands much more effective and potent antibacterial agents, hence, complexes outperform the ligands in terms of activity. The results show that Cu (II), Fe (III), and VO^2+^ complexes inhibited the growth of the selected bacteria and fungi the most. On the other hand, the Co(II) and Ni(II) complexes demonstrated modest activity. According to available data, binuclear complexes outperform acyclic complexes in terms of antibacterial activity, demonstrating that all these complexes are physiologically more efficient and implying that the chemical geometry of compounds is significant in explaining the complexes’ biological activity.

Badami et al. prepared Schiff base (HL) from 8-formyl-7-hydroxy-4-methylcoumarin and benzylamine, which they subsequently used to synthesize complexes of Co(II) (**13**), Ni(II) (**14**), and Cu(II) (**15**) (Figure 4) [33]. The Schiff base metal complexes were investigated for their antibacterial (*E. coli*, *P. aeruginosa*, *Klebsiella pneumoniae* (*K. pneumoniae*), and *S. aureus*) and antifungal (*Penicillium chrysogenum* (*P. chrysogenum*), and *Aspergillus niger* (*A. niger*)) activities, and it was discovered that the metal complexes had a more deadly impact on bacteria and fungus development than their parent ligand (Table 1). Against all strains, compound **15** demonstrated potential antibacterial and antifungal action. Solubility, conductivity, dipole moment, and cell permeability factors are thought to be the probable causes for the enhancement in the activity.

In another report, Schiff base ligand 3-chloro-N-((7-hydroxy-4-methyl-2-oxo-2H-chromen-8-yl)methylene)benzo[b]thiophene-2-carbohydrazide and its Cu(II) (**16**), Co(II) (**17**), Ni(II) (**18**), and Zn(II) (**19**) (Figure 4) complexes with octahedral geometries were synthesized by Mruthyunjayaswamy et al. [34]. The compounds’ antibacterial activity was evaluated in vitro against two Gram-negative bacteria (*E. coli* (MTCC 46) and *Salmonella typhi* (*S. typhi*) (MTCC 98)) and two Gram-positive bacteria (*B. subtilis* (MTCC 736) and *S. aureus* (MTCC 3160)) (Table 1). *C. albicans* (MTCC 227), *Cladosporium oxysporum* (*C. oxysporum*) (MTCC 1777), and *A. niger* (MTCC 1881) (Table 1) were tested for antifungal activity in vitro. All of the synthesized compounds showed antimicrobial capabilities in antimicrobial screening, and as expected, the metal complexes exhibited a greater inhibitory impact than their parent ligands and metal chlorides. Apart from the chelation theory, the increase in activity can be explained by the fact that most ligands have an azomethine link. Additionally, in metal complexes the positive charge of the metal ion is shared partially with the hetero donor atoms found in the ligand, and p-electron delocalization may occur throughout the chelating system. As a result, increasing the lipophilic character of metal chelates facilitates their passage through the lipoid layer of bacterial membranes and the blockage of metal binding sites in microorganism enzymes. Other factors that promote activity include conductivity, solubility, and the length of the link between the ligand and the metal ions.

From the coumarin–imine ligand, 8-[(1E)-1-(2-aminophenyliminio)ethyl]-2-oxo-2H-chromen-7-olate, Al-Amri et al. synthesized a series of metal complexes of Zn(II) (**20**), Ni(II) (**21**), Cu(II) (**22**), Pd(II) (**23**), and Cd(II) (**24**) (Figure 4) [35]. Compound **22** had the maximum efficacy and the lowest inhibitory concentration on the enzymatic activities of the investigated microbial species (Table 1). The agar plate antifungal activity of coumarin imine ligand and its metal complexes was evaluated against eight plant pathogenic fungi species (*Alternaria alternata* (*A. alternate*), *A. flavus*, *Botrytis cinerea* (*B. cinerea*), *Fusarium verticillioides* (*F. moniliforme*) and *Verticillium albo atrum* (*V. alboatrum*)) (Table 1). The copper complex outperformed the three most commonly used antifungal drugs. It had 100% radial inhibition (RI) against the most susceptible fungus, *A. flavus*. The RI of the tetrahedral cadmium complex against *A. alternata*, *A. flavus*, *F. moniliforme*, and *V. alboatrum* was 90%. However, the octahedral **20** possessed a 95% RI against B. cinerea. The square planar **23** has antifungal activity of 57–80%, with the highest activity against *A. flavus* and *F. moniliforme*. The octahedral **21** had the least antifungal activity, comparable to the drug Nystatin. The antibacterial activity of the investigated compounds was evaluated against four Gram-positive bacteria (*S. citrus*, *Streptococcus pneumoniae* (*S. pneumoniae*), *B. subtilis*, and *Micrococcus luteus* (*M. luteus*)) and four Gram-negative bacteria (*Enterobacter aerogenes* (*E. aerogenes*), *E. coli*, *P. aeruginosa*, and *S. typhi*). Even though the complexes under examination stopped bacteria from growing, they had a stronger antibacterial impact against Gram-positive bacteria than Gram-negative strains. Also, antibacterial activity was lowest for **21**. The antibacterial and antifungal activities increased with metal chelation. The square planar copper complex has excellent antibacterial activity, which can be attributed to its structure.

Linert et al. prepared two new mono and binuclear complexes with a Schiff base ligand obtained from the condensation of 3-acetylcoumarine and diethylenetriamine [36]. With cobalt(II), nickel(II), copper(II), zinc(II), and oxovanadium(II), the Schiff base ligand formed mono- or bi-nuclear cyclic or macrocyclic complexes, depending on the metal-to-ligand mole ratio and preparation method (**25**–**34**) (Figure 4). The Schiff base, HL, ligand, and its metal complexes were investigated for antibacterial activity against Gram-positive and Gram-negative bacteria, as well as a pathogenic fungus, *Fusarium oxysporum* (*F. oxysporum*) (Table 1). The findings were compared with the gram-negative and gram-positive antibiotics, chloramphenicol and cephalothin. The antifungal standard was cycloheximide. In vitro antibacterial and antifungal activity showed that complexes outperform ligands. The π-electron delocalization and partial sharing of the positive charge with a donor group lower the metal atoms polarity and this chelation might increase the metal atom’s lipophilicity, allowing it to pass through the lipid layers of the cell membrane and block metal binding sites on enzymes. The results showed that Cu(II) complexes had the greatest growth inhibition against Gram-positive bacteria, Gram-negative bacteria, and fungi. Complexes of Co(II), Ni(II), Zn(II), and VO(IV) had modest activity. The research demonstrated that binuclear macrocyclic properties improve antibacterial activity rather than acyclic complexes.

Badami et al. synthesized Co(II), Ni(II), and Cu(II) metal complexes from 6-formyl-7,8-dihydroxy-4-methylcoumarin and o-toluidine/3-aminobenzotrifluoride (**35**–**40**) (Figure 4) [37]. The compounds were evaluated for antibacterial (*E. coli* and *P. aeruginosa*), and antifungal (*Candida*, *A. niger*, and *Rhizopus*) activities using the disc diffusion technique (Table 1). Only a few species had antibacterial activity, and **40** had the most. Compounds **39** and **40** outperformed the others in antifungal activity. Protein synthesis is an important phase in the development of microorganisms. Metal ions function as growth inhibitors for microorganisms by adsorbing on the outside surface of the cell wall and preventing respiration. This interrupts protein synthesis and kills the microorganism. The increased activity of chelates may be owing to the metal ion’s decreasing polarity due to sharing its positive charge with donor groups, as explained by chelation theory.

Kinni et al. have synthesized metal complexes of type ML_2_, where M = Co(II), Ni(II), Cu(II), Cd(II), Zn(II), Hg(II), and L = Schiff base (**41**–**46**) (Figure 4) [38]. *E. coli*, *S. aureus*, *B. subtilis*, *P. aeruginosa* bacteria and *A. flavus*, *A. niger*, *C. oxysporum*, and *C. albicans* fungal strains were used to test the antibacterial and antifungal capabilities of the synthesized compounds by the minimum inhibitory concentration (MIC) method (Table 1). Coordination with metal ions clearly increased the ligand’s efficacy and also lessened the metal ion’s polarity by sharing its positive charge with donor groups inside the chelate ring system generated during coordination. Because of its high lipophilicity, the central metal atom may easily penetrate the lipid layer of bacteria, causing them to die.

Bajroliya et al. developed Mn(II) and Co(II) metal complexes from novel Schiff bases derived from 8-formyl-7-hydroxy-4-methylcoumarin and 3-substituted 4-amino-5-mercapto-1,2,4-triazole by using microwave irradiation as well as conventional methods (**47**–**56**) (Figure 4) [39]. Compared with the traditional approach, the microwave irradiation procedure yielded higher yields with better selectivity. This study investigated the in vitro antimicrobial activity of synthesized Schiff bases and their metal complexes by measuring the zone of inhibition in mm. Assays were performed using gentamycin at a 100 g/mL concentration against five species of bacteria: *E. coli*, *P. aeruginosa*, *S. typhi*, *S. aureus*, and *B. subtilis*. Antifungal activities were assessed against *A. niger* and *C. albicans* at 100 g/mL using fluconazole as a reference drug. The metal complexes of all Schiff bases had stronger antibacterial activity than the Schiff bases alone against selected bacteria and fungi (Table 1). The Mn(II) and Co(II) metal complexes showed significantly increased activity against *S. typhi*. The Schiff bases, as well as their Mn(II) and Co(II) complexes, were found to have good antifungal activity. Al-Amiery et al. prepared novel transition metal complexes [M(L_1_)_2_Cl_2_] and [M(L_2_)_2_Cl_2_] by reacting MCl_2_.nH_2_O (M = Co, Ni, Cu) with (Z)-1-(1-(1H-indol-3-yl)ethylideneamino)quinolin-2(1H)-one (L_1_) or (E)-1-(2-hydroxybenzylideneamino)quinolin-2(1H)-one (L_2_) (**57**–**62**) (Figure 4) [40]. The antibacterial activity of the prepared complexes was then estimated against *S. aureus* (Gram positive) and *E. coli* and *P. aeruginosa* (Gram negative) bacteria (Table 1). The enhanced activity of metal complexes can be explained by the overtone concept and chelation theory. Liposolubility is a key element in determining antimicrobial action since it allows only lipid-soluble molecules to get through the lipid barrier surrounding the cell. Chelation reduces the polarity of the metal ion due to ligand orbital overlap and partial sharing of the positive charge with donor groups. It also boosts the lipophilicity of the complex and the delocalization of electrons across the chelate ring. This enhanced lipophilicity promotes complex penetration through the lipid membrane and prevents metal binding sites on the microorganism’s enzymes.

Dhumwad et al. prepared complexes of Co(II), Ni(II), Cu(II), and Zn(II) with Schiff bases of N′-[(E)-(2-hydroxyquinolin-3-yl)methylidene]-2-[(4-methyl-2-oxo-2H-chromen-7-yl)oxy] acetohydrazide (OHQZ) and 2-[(4-methyl-2-oxo-2H-chromen-7-yl)oxy]-N′-[(E)-(2-sulfanylquinolin-3-yl)methylidene] acetohydrazide (SHQZ) (**63**–**70**) (Figure 4) [41]. The antibacterial and antifungal properties of the synthesized Schiff bases and their Co(II), Ni(II), Cu(II), and Zn(II) complexes were investigated using the potato dextrose agar diffusion method and the nutritional agar method. The minimum inhibitory concentration (MIC) technique was used to test the antibacterial and antifungal activities on two bacterial (*E. coli* and *S. aureus*) and two fungal (*A. niger* and *P. chrysogenum*) strains (Table 1). Based on the in vitro antibacterial and antifungal activity against selected bacterial and fungal strains, it is clear that the Cu(II) complexes are more active at lower MIC values for bactericidal activity. Badami et al. prepared a series of metal complexes of the type ML·2H_2_O (M = Co(II), Ni(II), and Cu(II)) with Schiff bases produced from 1,8-diaminonaphthalene and 8-formyl-7-hydroxy-4-methylcoumarin/8-acetyl-7-hydroxy-4-methylcoumarin (**71**–**76**) (Figure 4) [42]. The MIC method was employed to determine the antibacterial (*E. coli*, *S. aureus*, *P. aeruginosa*, and *S. typhi*) and antifungal (*A. niger*, *A. flavus*, and *Cladosporium*) properties of the Schiff bases and their complexes (Table 1). In antibacterial experiments, the Schiff bases were found to be potentially active against *E. coli* and *S. typhi*, as well as moderately active against *S. aureus*. Compounds **71** and **72** had substantial antibacterial activity against *E. coli*, *P. aeruginosa*, and *S. typhi* and least activity against *S. aureus*. Compounds **73** and **74** were effective against *E. coli*, *S. aureus*, and *S. typhi*, but only modestly effective against *P. aeruginosa*. Compounds **75** and **76** had outstanding antibacterial activity against *S. typhi* but only fair activity against *E. coli*, *P. aeruginosa*, and *S. aureus*. All Schiff bases were active against fungi. However, as compared with the uncoordinated compounds, **71**–**76** exhibited significantly increased activity, notably with A. flavus. The findings show that activity relies on metal ion type and varies in the following order: Cu > Ni > Co. 

**Figure 5:**
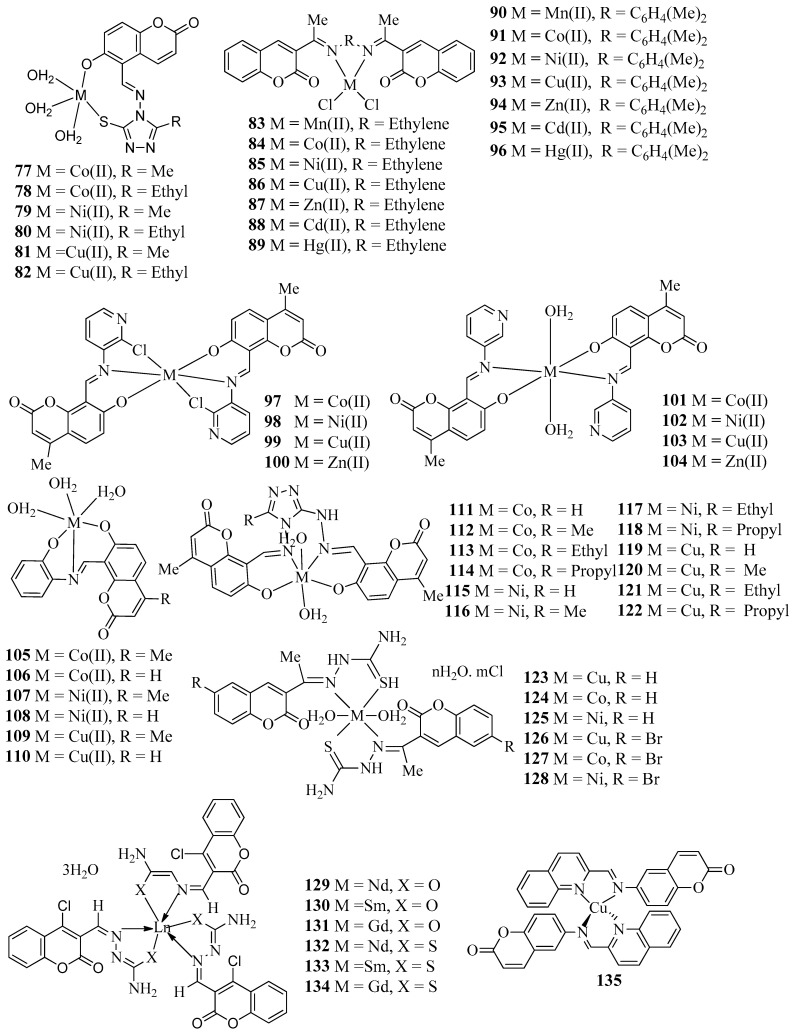

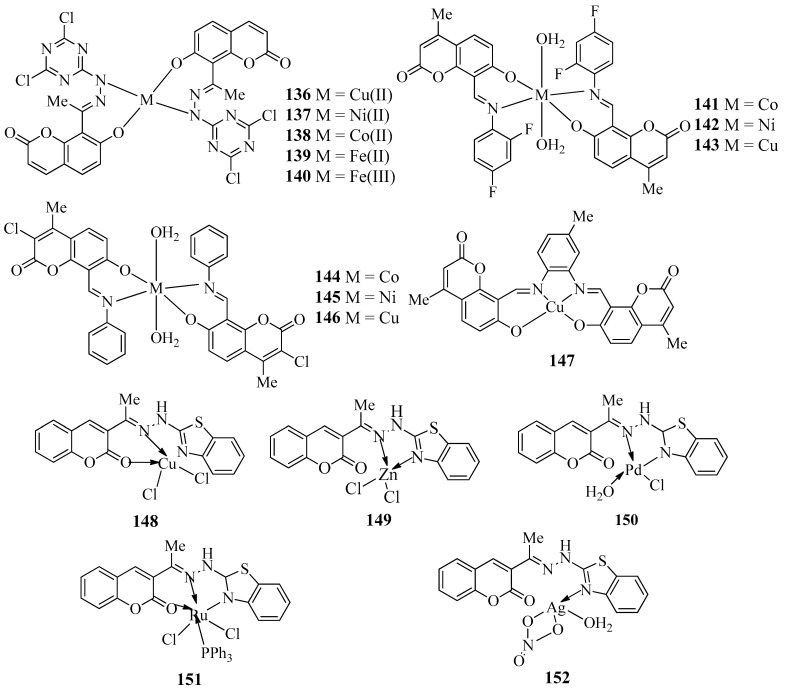
Structures of coumarin-derived imine–metal complexes (**77–152**).

Patil et al, in 2011 reported the synthesis of a series of Co(II), Ni(II), and Cu(II) complexes (**77**–**82**) (Figure 5) with Schiff bases derived from 3-substituted-4-amino-5-mercapto-1,2,4-triazole and 5-formyl-6-hydroxy coumarin [43]. All synthesized metal complexes (**77**–**82**) were evaluated for antibacterial activities against four bacterial species, *E. coli*, *S. aureus*, *P. aeruginosa*, and *S. typhi*, and antifungal efficacy towards three fungi, namely *A. niger*, *A. flavus*, and *Cladosporium* through the minimum inhibitory concentration method. All the metal complexes (**77**–**82**) showed promising antimicrobial activities against some bacterial and fungal strains. Among the prepared metal complexes, Co(II) complex **77** showed maximum efficacy against *P. aeruginosa* with MIC 10 µg/mL and Ni(II) complex **80** exhibited the highest antifungal activity against *Cladosporium* with MIC 10 µg/mL.

The metal complexes (**83**–**96**) (Figure 5) with Schiff bases derived from the condensation of 3-acetylcoumarine with ethylenediamine and orthophenylenediameine were developed by Akkasali et al. [44]. All the developed compounds (**83**–**96**) were screened for their possible antimicrobial activity against *E. coli*, *P. aeruginosa*, *A. niger*, and *C. albicans*. The prepared metal complexes (**83**–**96**) showed moderate to high activity against the tested organisms. Among the complexes, copper complex **93** showed a maximum zone of inhibition against *P. aeroginosa* and nickel complexes **85** and **92** displayed higher antifungal activity against *A. niger*. In addition, nickel complex **92** also revealed good activity against *C. albicans*. However, all the developed compounds were less active than the standard drugs ciprofloxacin and fluconazole and the data presented in this review could be a helpful guide for medicinal chemists who are working in the area. Dhumwad and co-workers have designed the synthesis of metal complexes (**97**–**104**) (Figure 5) of Schiff bases formed through the condensation of 8-formyl-7-hydroxy-4-methyl-coumarin with 3-amino-pyridine and 3-amino-2-chloro-pyridine [45]. The synthesized metal complexes (**97–104**) were evaluated for their in vitro antibacterial potential against *S. aureus* and *E. coli*, and antifungal efficacy against two fungi, viz., *A. niger* and *C. albicans*. Among the metal complexes, copper complexes **99** and **103** are found to be most active against the tested bacteria with a MIC value of 1.56 µg/mL, which is almost equal to that of the standard drug, norfloxacin. Therefore, the prepared copper complexes **99** and **103** are found to be more effective bactericides than fungicides.

Patil et al. have structured and developed a new class of Co(II), Ni(II), and Cu(II) complexes (**105**–**110**) (Figure 5) with Schiff bases derived from 8-formyl-7-hydroxy-4-methylcoumarin or 5-formyl-6-hydroxycoumarin and *o*-aminophenol and assessed them for their in vitro antibacterial action against microorganisms such as *E. coli*, *P. aeruginosa*, *S. typhi*, *A. flavus and Cladosporium*, *A. flavus*, *Cladosporium*, *and A. niger* (Table 1) [46]. From the MIC studies, it has been found that the metal complexes were more active against both bacterial and fungal species than parent ligands. Among the metal complexes, cobalt complex **105** and nickel complex **107** revealed high activity against *E. coli*, *P. aeruginosa*, and *S. typhi.* Furthermore, cobalt complex **105** and nickel complex **107** also had good antifungal activity against *A. flavus*, *Cladosporium*, and *A. niger*. Patil et al. have also designed and synthesized a series of cobalt(II), nickel(II), and copper(II) complexes (**111**–**122**) (Figure 5) with newly derived biologically active ligands that are prepared from the condensation of 3-substituted-4-amino-5-hydrazino-1,2,4-triazole and 8-formyl-7-hydroxy-4-methylcoumarin [47]. All the metal complexes (**111**–**122**) were evaluated for their antibacterial efficacy against four bacterial strains, namely *E. coli*, *S. aureas*, *S. pyogenes*, and *P. aeruginosa* using Gentamycin as the standard. From the antibacterial studies, it was found that all the synthesized metal complexes (**111**–**122**) were more active compared with the corresponding parent Schiff bases. Metal complex **116** showed maximum inhibition against *S. aureas* and *P. aeruginosa* at 25 µg/mL. Furthermore, metal complex **118** showed maximum inhibition towards *E. coli* at 25 µg/mL. Also, metal complex **120** displayed highest inhibition against *P. aeruginosa* at 25 µg/mL, whereas metal complex **121** revealed maximum inhibition towards *S. aureas* and *S. pyogenes* at 25 µg/mL. The synthesized metal complexes **111**–**122** (Table 1) were also screened for their antifungal activities against *A. Flavus*, *Cladosporium*, and *A. niger* using the standard drug fluconazole. Metal complex **114** presented MIC 10 µg/mL against *S. aureus* and *A. Niger*, whereas metal complex **120** showed MIC 10 µg/mL against *A. Flavus*.

A series of Schiff base complexes of Cu(II), Co(II), and Ni(II) (**123**–**128**) (Figure 5) with two coumarin-3-yl thiosemicarbazone derivatives (1E)-1-(1-(2-oxo-2H-chromen-3-yl)ethylidene)thiosemicarbazide and (1E)-1-(1-(6-bromo-2-oxo-2H-chromen-3-yl)ethylidene)thiosemicarbazide were constructed and tested for their in vitro antibacterial potential towards both Gram-negative and Gram-positive bacterial species such as *E. coli*, *P. aeruginosa*, *B. subtili*, and *S. aureus by* Refat et al. [48]. From the in vitro antibacterial evaluation, it was found that all the synthesized metal complexes **123**–**128** showed more efficacy than their parent Schiff base ligands against both Gram-negative and Gram-positive bacterial species such as *E. coli*, *P. aeruginosa*, *B. subtili*, and *S. aureus.* Conversely, metal complexes **124** and **128** exhibited maximum inhibition zone against *B. subtili*. Similarly, metal complexes **124** and **127** exhibited maximum inhibition zone against *S. aureus*, whereas metal complex **127** displayed maximum inhibition zone against *E. coli*. Kapoor et al, in 2012 explored the microwave-assisted synthesis and biological evaluation of coumarin-based lanthanide complexes (**129**–**134**) (Figure 5) [49]. All complexes (**129**–**134**) were successfully synthesized using rare earth metals Nd(III), Sm(III), and Gm(III) with ligands 3-formyl-4-chlorocoumarin hydrazinecarboxamide and 3-formyl-4-chlorocoumarinhydrazine carbothioamide. All the lanthanide complexes **129**–**134** were evaluated for their antimicrobial activity against four bacteria (*E. coli*, *P. aeruginosa*, *B. subtilis*, and *S. aureus*), and three fungi (*C. albicans*, *A. niger*, and *F. oxysporum*). From the results, it has been found that complexes showed more activity compared with the paternal Schiff base ligands and were also compared against standard drugs ciprofloxacin for bacterial species and fluconazole for fungal species. All the aforementioned lanthanide complexes (**129**–**134**) showed MIC values in between 10–40 MIC (µg/mL). In particular, Lanthanide complex **130** displayed MIC 10 µg/mL against *E. coli*, *S. aureus*, *C. albicans*, *A. niger*, and *F. oxysporum* w, 15 µg/mL against *P. aeruginosa*, and 20 µg·mL^−1^ against *B. subtilis.* Conversely, complex **131** showed 84 ± 0.6% inhibition against *F. oxysporum* at 100 ppm, whereas complex **129** showed 78 ± 0.7% inhibition against *C. albicans* at 100 ppm (Table 1).

In the recent past, Modak et al. reported the synthesis of copper complex **135** (Figure 5) with imine ligand 6-((quinolin-2-ylmethylene)amino)-2H-chromen-2-one achieved from derivatization of natural compound coumarin [50]. The potential antibacterial effect of copper complex **135** was assessed for *Flavobacterium psychrophilum* (*F. psychrophilum*) isolated 10094 and efficacy was IC_50_ 16.1 ± 0.9 µg/mL. However, MIC and MBC of the copper complex **135** were found to be lower (32 µg/mL) than the precursor (64 µg/mL). Novel classes of metal complexes (**136**–**140**) (Figure 5) that are derived from 7-hydroxy coumarin hydrazone of *s*-triazine derivatives were evaluated for their in vitro antibacterial potency against tested pathogens by the research group of Jani [51]. Antibacterial activity was tested using the agar diffusion method against *E. coli* and *S. aureus* and *A. niger* was used for antifungal activity. From the zone of inhibition data, it was concluded that metal complex **136** exhibited higher inhibition towards *S. aureus*, *E. coli*, *Aspergillus niger*, and *S. pyogenes*, whereas the other metal complexes **136**–**140** revealed moderate activity. All metal complexes (**136**–**140**) were inactive against *P. klebsiella* (Table 1).

In 2016, Patil and co-workers reported the synthesis of a series of Co(II), Ni(II), and Cu(II)complexes (**141**–**146**) (Figure 5) from the reaction 8-formyl-7-hydroxy-4-methylcoumarin/3-chloro-8-formyl-7-hydroxy-4-methylcoumarin with 2,4-difluoroaniline/o-toluidine [52]. Antimicrobial activities of metal complexes **141**–**146** were carried out through the disc diffusion method against two bacterial strains (*P. auregenosa* and *Proteusmirabilis*) with gentamicin as the standard and two fungal strains (*A. niger* and *R. oryzae*) with antifungal drug fluconazole (Table 1). Among different halogenated metal complexes, copper complex **146** exhibited more activity against *A. niger.* Nickel complex **145** displayed a maximum zone of inhibition against *P. auregenosa*, whereas copper and nickel complexes **143**, **145**, and **146** revealed maximum inhibition against *Proteusmirabilis*. Copper complex **147** (Figure 5) with a salen-type ligand derived from the condensation of 4-methyl-7-hydroxy-8-formylcoumarin with 3,4-diaminotoluene was screened for in vitro antibacterial activity by Sharma et al. through the broth dilution method against four bacterial strains: *E. coli*, *S. aureus*, *P. aeruginosa*, and *K. pneumonia* [53]. Compound **147** exhibited an MIC of 12.5-200 μg·mL^−1^ and appeared to be active against both Gram-positive and Gram-negative bacteria. Both experimental and theoretical calculations revealed that **147** has better antibacterial potential than the parent Schiff base ligand (Table 1).

A series of five new Cu (II), Zn (II), Pd (II), Ru (III), and Ag(I) complexes (**148**–**152**) (Figure 5) derived from the 3-acetylcoumarin-2-hydrazinobenzothiazole Schiff base have been synthesized and characterized by Atlam et al. [54]. All metal complexes **148**–**152** were screened for their antifungal effect towards two fungal strains, *G. recinaceum* (GR33) and *O. latemarginatus* (EM26). Palladium complex **150** acts as the best antifungal agent for GR33 with a 60% reduction, whereas the silver complex **152** has a 79% reduction for *O. latemarginatus* (EM26). The ligninolytic activity of the complexes on fungi was found using Poly R-dye decolorization ability. Results suggest that complexes showed decolorization at lower concentrations depending on the fungi used. From the observed results, it can be concluded that both palladium and silver complexes **152** and **150**, respectively, were used for antifungal preservatives, whereas copper, zinc, and ruthenium complexes **148, 149**, and **151**, respectively, can be used for lignolytic activity. Observed results were confirmed using computational studies and molecular docking studies help to provide a better understanding of binding interactions (Table 1).

**Figure 6:**
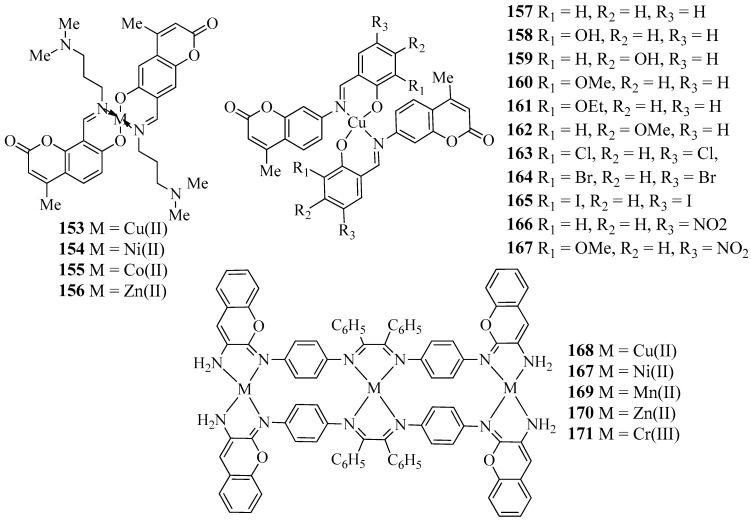
Structures of coumarin-derived imine–metal complexes (**153**–**171**).

Sawant et al. reported the synthesis, characterization and antimicrobial activity of novel transition metal complexes of 4-methyl-7-hydroxy 8-formyl coumarin. The prepared Schiff base acts as a bidentate ligand for the complexation with Cu(II), Ni(II), Co(II), and Zn(II) ions. The Schiff base and their transition metal complexes (**153**–**156**) (Figure 6) were screened for their antimicrobial activity towards two strains, *E. coli* and *A. niger*, through the tube dilution method in order to examine the effect of metal ions upon chelation (Table 1). All the transition metal complexes **153**–**156** showed MIC values less than 20 µg/mL compared with the ligand, which has MIC values less than 200 µg/mL against both species. The results of antimicrobial activity indicate that the antimicrobial activity of the Schiff base was increased on complexation with metal ions [55]. In 2009, Creaven and co-workers reported the Cu(II) complexes (**157**–**167**) (Figure 6) of coumarin-derived Schiff bases. A few of the Cu(II) complexes were characterized through X-ray crystallography technique to determine the structures. Furthermore, all Cu(II) complexes **157**–**167** were screened for their anti-*Candida* activity. All Cu(II) complexes displayed excellent anti-*Candida* activity comparable to that of commercially available drugs such as ketoconazole and amphotericin B (Table 1) [56]. 

Recently, Chandrasekaran et al. described the synthesis, characterization, and antibacterial activity of new Cu (II), Ni (II), Mn (II), Zn (II), and Cr (III) complexes (**168**–**171**) (Figure 6) of Schiff bases derived from *p*-phenylenediamine, benzil, and 3-aminocoumarin. Structural analyses of all new transition metal complexes **168**–**171** were conducted through infrared, ultraviolet–visible, cyclic voltammetry, EPR, magnetic property, and thermogravimetric techniques. Furthermore, all transition metal complexes **168**–**171** were tested for their antibacterial activity against bacterial species such as *K. pneumoniae*, *E. coli*, *and S. aureus* by the disc diffusion method. The biological activity results concluded that the Schiff base transition metal complexes **168**–**171** have more efficiency compared with standard streptomycin drug and the transition metal complexes **168**–**171** arrested the growth of bacterium (Table 1) [57]. 

### 3.2. Anticancer Activity of Coumarin-Derived Imine–Metal Complexes 

**Figure 7:**
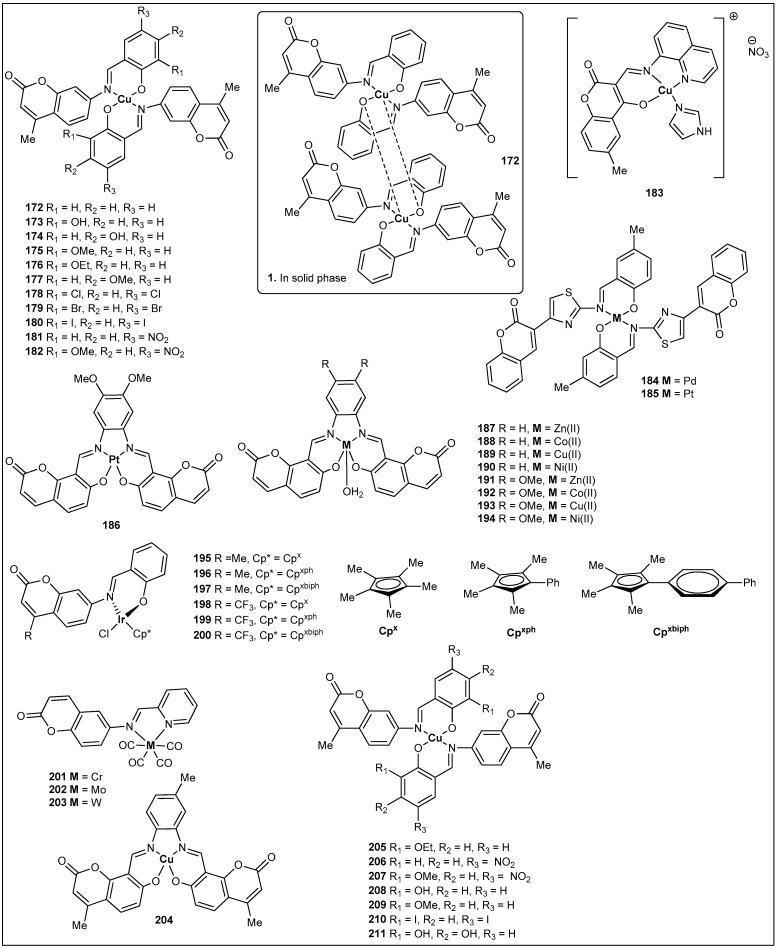
Structures of coumarin-derived imine–metal complexes (**172**–**211**).

It is well known that coumarin derivatives present important cytotoxic activity against several cell lines [18]. It is also known that coordination of bioactive molecules to transition metals display enhanced inhibitory activity [58]. In this regard, Aazam and coworkers prepared the first coumarin-derived imine–metal complex that has been evaluated for its anticancer activity. This complex was synthesized from the reaction of 4-methyl-7-(salicylideneamino)coumarin with Cu(OAc)_2_. Interestingly, single crystal X-ray diffraction studies exhibited that the complex crystallized as a dimeric copper coumarin complex, **172**, as depicted in Figure 7 [59]. In 2009, Creaven and coworkers corroborated this observation when they also observed the same dimeric copper complex [57]. Nonetheless, Creaven and coworkers went further and prepared a total of eleven coumarin copper complexes (**172**–**182**) (Figure 7) (Table 2). Although, other complexes were crystallized (e.g., **179**), only complex **172** was observed as dimeric copper complex [60]. A year later, Creaven et al. reported the cytotoxicity activity of the eleven complexes against two human cell lines: human colon cancer cells (HT29) and human breast cancer cells (MCF-7) using the methylthiazolyldiphenyl-tetrazolium bromide assay (MTT) [60]. Unfortunately, none of the complexes were cytotoxic against colon cancer cells and only complexes **180** and **182** showed cytotoxicity against breast cancer cells, with IC_50_ values of 79.8 μM and 34.5 μM, respectively. Values that are comparable with the positive control mitoxantrone (IC_50_ value of 44 μM) (Table 2). In an attempt to try to increase the observed cytotoxicity, the authors reduced the coumarin shift bases to amines. Sadly, their respective copper complexes were not successfully synthesized [60]. 

In 2017, Tabassum et al. reported the synthesis of one coumarin-derived imine–metal complex, **183** (Figure 7), that has an appended imidazole (Figure 4) [59]. In vitro anticancer studies revealed that complex **183** has superior cytotoxicity towards the A549 adenocarcinoma cell line in comparison with the standard drug cisplatin (Table 2), which is a very promising discovery as A549 is a cisplatin-resistant cell line [61] (Table 2). The same year, Sahin and coworkers reported the synthesis, characterization, and anticancer activity of two coumarin-derived imine–metal complexes. Synthesis of complex **184** (Figure 7) used Na_2_PdCl_4_ to incorporate palladium to the ligand, whereas K_2_PtCl_4_ was used to install platinum into complex **185** (Figure 7) [62]. Both complexes were evaluated against three human cancer cell lines: prostate cancer cells (ATTC), colon carcinoma cells (LNCap), and breast cancer cells (MCF-7) using the MTT assay. The data from the in vitro antitumor activity showed that palladium complex **184** had the most prominent activity against the three screened cell lines, with an IC_50_ of 18.15 μg/mL for MCF-7, an IC_50_ of 10.05 μg/mL for LNCap, and an IC_50_ of 15.98 μg/mL for ATTC. Values that are 3- to 5-fold better than the ligand starting material [60].

**Table 2:** Anticancer activity data of coumarin-derived imine–metal complexes.

Compound	Cell Line	IC_50_ (µM)	Ref.	Compound No.	Cell Line	IC_50_ (µM)	Ref.
**172**	MCF-7	no reported	[58]	**187**	HeLa	86.9 ± 9.0	[63]
**173**	MCF-7	no reported	[58]	**188**	HeLa	3.5 ± 1.2	[63]
**174**	MCF-7	no reported	[58]	**189**	HeLa	52.5 ± 1.0	[63]
**175**	MCF-7	no reported	[58]	**190**	HeLa	72.7 ± 8.1	[63]
**176**	MCF-7	no reported	[58]	**191**	HeLa	90.7 ± 2.5	[63]
**177**	MCF-7	no reported	[58]	**192**	HeLa	4.1 ± 0.9	[63]
**178**	MCF-7	no reported	[58]	**193**	HeLa	83.5 ± 4.7	[63]
**179**	MCF-7	no reported	[58]	**194**	HeLa	85.1 ± 1.6	[63]
**180**	MCF-7	79.8 ± 5	[58]	**Cisplatin**	HeLa	11.6 ± 2.7	[63]
**181**	MCF-7	no reported	[58]	**195**	A549	30.9 ± 1.6	[63]
**182**	MCF-7	34.5 ± 6	[58]	**196**	HeLa	9.9 ± 0.1	[64]
**Mitoxantrone**	MCF-7	44 ± 3	[58]	**197**	HeLa	10.8 ± 0.1	[64]
**183**	A549	4.6 ± 0.3	[59]	**198**	A549	39.6 ± 1.0	[64]
**Cisplatin**	A549	57.7 ± 0.9	[59]	**199**	HeLa	22.1 ± 0.5	[64]
**184**	LNCap	10.05	[59]	**200**	A549	33.4 ± 0.7	[64]
**185**	LNCap	21.53	[59]	**Cisplatin**	A549	21.3 ± 1.7	[64]
**186**	HeLa	61.4 ± 1.1	[59]	**Cisplatin**	HeLa	7.5 ± 0.2	[64]

In 2020, Hurtado et al. reported nine tetradentate coumarin Schiff-based metal complexes (**186**–**194**) (Figure 7) [63]. Two coumarin ligands and the five metals (Zn(OAc)_2_, Co(OAc)_2_, Cu(OAc)_2_, Ni(OAc)_2_, and K_2_PtCl_4)_ were used to prepare the nine complexes (Figure 4). A cytotoxicity activity was carried out using an MTT assay in the carcinogenic cell line HeLa (human cervical cancer cells), and, to make the study more relevant, two noncarcinogenic cell lines, HFF-1 (human foreskin fibroblast cells) and HaCaT (human keratinocytes) were screened. Data from this study showed that both ligands and seven of the screened complexes displayed higher IC_50_ values than the standard drug cisplatin (IC_50_ = 11.6 μM). Fortunately, both cobalt complexes **188** and **192** exhibited lower IC_50_ values, or in other words the highest cytotoxicity towards HeLa cells. For instance, complex **188** showed an IC_50_ = 3.5 μM and complex **192** showed an IC_50_ = 4.1 μM (Table 2). Thus, they have promising anticarcinogenic potential [64]. The same year, Liu et al. published the synthesis, characterization, and anticancer activity of six coumarin-derived imine–iridium complexes (**195**–**200**) (Figure 7) [64]. The MTT assay was used to determine the anticancer activity of the former stated complexes. The studied human cancer cell lines were lung cancer cells (A549) and cervical cancer cells (HeLa) with cisplatin as the positive control. All complexes displayed IC_50_ values ranging from 9.9 μM to 40.7 μM, with complex **196** showing almost double (12 μM) the anticancer activity of cisplatin (21.3 μM) against lung cancer cells (Table 2) [64]. It is important to note that fluorinated complexes showed similar or lower antitumor activity.

### 3.3. Antioxidant Activity of Coumarin-Derived Imine–Metal Complexes 

Following similar methodology as mentioned above, three new coumarin-derived imine–metal complexes were synthesized by Sinha et al. from the condensation of 6-aminocoumarin and pyridine-2-carboxaldehyde to make a ligand [65]. The ligand was reacted with Cr(CO)_4_, Mo(CO)_4_, and W(CO)_4_ to produce complexes (**201**–**203**) (Figure 7). The three complexes were tested for their antioxidant properties and their antioxidant activity (radical scavenging activity) was examined with reference to 1,1-diphenyl-2-picrylhydrazyl (DPPH), hydroxyl radical (OH·), superoxide(O_2_^−^), and nitroxyl radical (NO·). The data revealed that the complexes had a maximum percentage of inhibition at concentrations of 0.03 mg/mL, 0.3 mg/mL, and 0.01 mg/mL for [Cr(CO)_4_(L)], [Mo(CO)_4_(L)], and [W(CO)_4_(L)], respectively. Therefore, complex **203**, having the heaviest element (W), shows the highest scavenging ability. In 2012, Halli and coworkers reported another series of coumarin-derived imine–metal complexes (**41**–**46**) (Figure 4) [38]. In addition to the antimicrobial activity (reported above), an antioxidant assay (DPPH) was investigated for all the synthesized complexes, and the tabulated data in Table 3 shows that the top radical scavengers were complexes **42** (Ni), **44** (Cd), and **46** (Hg) (Table 3) at a concentration of 100 μg/mL [66]. Similarly, Sharma et al. reported the synthesis, antibacterial, and antioxidant activity of complex **204** (Figure 7) [67]. Although complex **39** presented some scavenging ability (30%), it was lower in comparison with the standards gallic acid (90%) and quercetin (80%) at a concentration of 62.5 μg/mL. Finally, Creaven’s group has also studied a series of coumarin-derived imine–copper complexes (**205**–**211**) (Table 3) (Figure 7) [66]. Besides cytotoxicity studies (data not relevant) of those complexes, their antioxidant activity was studied in wild type cells (*Saccharomyces cerevisiae*). Interestingly, the survival rates for wild-type cells exposed to oxidative stress (with menadione) was excellent. For instance, wild-type cells treated with complexes **205**–**211** had a typical survival of around 90%, versus 15% for untreated cells. However, when the oxidative stress was induced with H_2_O_2_, none of the complexes were able to protect the cells, perhaps due to the lack of CAT enzymes in the selected wild-type cells [68]. In summary, it is safe to say that all the coumarin-derived imine–metal complexes present some scavenger activity, and that it is always superior to that of their respective coumarin ligands [68]. 

### 3.4. Anthelmintic Activity of Coumarin-Derived Imine–Metal Complexes

Drugs that are used to treat the infections of animals with parasitic worms are called anthelmintics. In 2015, Badami et al. reported the coumarin-derived imine Co(II), Ni(II), and Cu(II) complexes, **13**–**15** (Figure 4), for anthelmintic (*Pheretima posthuma*) activity study. On the basis of the obtained results, it can be considered that metal complexes are more active than their parent ligands. Predominantly, Cu(II) complex **15** exhibited excellent activity, compared with the standard drug, albendazole, at a 10 μg/mL concentration (Table 4) [33]. In the same year, Badami et al. also investigated anthelmintic activity of the Schiff bases metal complexes, **35**–**40** (Figure 4), on adult Indian earthworm, *Pheretima posthuma*. All the metal complexes **35**–**40** exhibited potent anthelmintic activity compared with the standard drug (albendazole) (Table 4). Specifically, metal complexes **37** and **40** displayed noticeable activity compared with the standard drug, albendazole, at a 10 µg/mL concentration [37].

### 3.5. Pesticidal and Nematicidal Activity of Coumarin-Derived Imine–Metal Complexes

Pests and nematodes inhabit numerous trophic levels and together disturb the entire ecological balance. They grow for centuries, causing diverse infections notoriously lethal to plants, humans, and animals. Due to this ever-present issue, it is vital to identify sustainable effective nematicides and insecticides. In 2011, Fahmi et al. reported pesticidal and nematocidal activities of lanthanide(III) complexes (**129**–**131**) (Figure 5) with 3-formyl-4-chlorocoumarin hydrazinecarbothioamide and 3-formyl-4-chlorocoumarin hydrazinecarboxamide against *Tribolium castaneum* and *Meloidogyne incognita*. All the metal complexes exhibited good pesticidal activity compared with their corresponding ligands. Sm(III) complex **130** was especially effective as evidenced through the comparative study of their percentage mortality data with other metal complexes (**129** and **131**). The nematocidal activity displays that metal complexes **129**–**131** were more active in lowering the hatching of eggs than the corresponding ligands [46].

### 3.6. Enzyme Activity of Coumarin-Derived Imine–Metal Complexes

**Figure 8:**
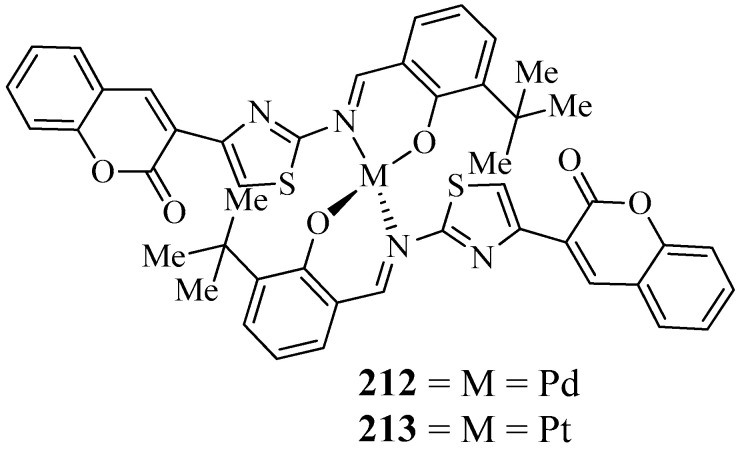
Structures of coumarin-derived imine–metal complexes (**212**, **213**).

For the treatment of Alzheimer’s disease (AD), cholinesterase inhibition is the only valid target used. Hence, there is a continuous need for the development and synthesis of new cholinesterase inhibitor molecules. Recently, Özdemir and co-workers reported palladium and platinum complexes derived from combining coumarin and thiazole with 3-tertiary butyl salicylaldehyde, a new Schiff base [67]. Metal complexes **212** and **213** (Figure 8) were structurally characterized from various spectroscopic techniques. Additionally, the authors investigated the inhibitor potencies of metal complexes on three esterase enzymes, acetylcholinesterase (AChE), butyrylcholinesterase (BChE), and pancreatic cholesterol esterase activities (CEase). The anti-esterase activity results directed that metal complexes have good inhibition effects on the cholinesterase (AChE/BChE) and pancreatic cholesterol (CEase) enzymes. Particularly platinum complex **213** exhibited a high inhibition effect with lower IC50 values (12.0 μM for AChE, 23 μM for BChE, and 21.0 μM for CEase) compared with the reference drugs [67].

## 4. Conclusions and Future Perspective

Searching through drug banks for coumarin-derived clinically approved agents has yielded several important drugs, including a treatment for HIV that combines several different compounds and includes calanolide A, a coumarin agent. Currently, a number of clinical trials are being conducted on several coumarin-based drugs alone or in combination with other drugs to treat common disorders, such as thrombosis, coagulation disorders, stroke (Ischemic), liver fibrosis, protein C deficiency, atrial fibrillation, etc. From these findings it can be seen that coumarin-based drugs have an important role in global health and well-being and are continuously researched to discover more clinical uses.

Coumarin scaffold has great potential in medicinal chemistry and is extensively used in drug design and development because of its vast biological properties. This scaffold is frequently used for designing small molecules with various biological activities. Metal complexes of Schiff bases with coumarin pharmacophores have played an important role in medicinal chemistry. Various coumarin-derived imine–metal complexes have been developed in the last three decades and most of them exhibited important pharmacological properties. 

## Figures and Tables

**Table 1:** Antimicrobial activity of coumarin-derived imine–metal complexes.

Compound	Bacteria Screened (n)	Fungi Screened (n)	Highest Activity Against	Concentration	Activity Inhibition Zone (mm)	Ref.
**1**	5	3	*B. subtilis*, *S. aureus*, *S. faecalis* and *P. aeruginosa*	NA	13	[31]
**2**	5	3	*S. aureus*	NA	11	[31]
**3**	2	1	*F. oxysporum*	NA	26 ± 0.2	[32]
**4**	2	1	*P. phaseolicola*	NA	28 ± 0.3	[32]
**5**	2	1	*S. aureus*	NA	36 ± 0.3	[32]
**6**	2	1	*S. aureus*	NA	33 ± 0.1	[32]
**7**	2	1	*S. aureus*	NA	37 ± 0.4	[32]
**8**	2	1	*S. aureus*	NA	27 ± 0.4	[32]
**9**	2	1	*F. oxysporum*	NA	29 ± 0.3	[32]
**10**	2	1	*S. aureus*	NA	31 ± 0.1	[32]
**11**	2	1	*F. oxysporum*	NA	34 ± 0.3	[32]
**12**	2	1	*S. aureus*	NA	32 ± 0.2	[32]
**13**	4	3	*E. coli*	200 μg/mL	NA	[33]
**14**	4	3	*S. aureus*	200 μg/mL	NA	[33]
**15**	4	3	*S. aureus*	200 μg/mL	NA	[33]
**16**	4	3	*B. subtilis*, *E. coli* and *A. niger*	12.5 μg/mL	MIC	[34]
**17**	4	3	*B. subtilis* and *C. oxysporum*	12.5 μg/mL	MIC	[34]
**18**	4	3	*B. subtilis*	12.5 μg/mL	MIC	[34]
**19**	4	3	*B. subtilis*, *E. coli*, *A. niger* and *C. oxysporum*	12.5 μg/mL	MIC	[34]
**20**	8	0	*B. subtilis*	NA	MP	[35]
**21**	8	0	*B. subtilis*	NA	MP	[35]
**22**	8	0	*B. subtilis*	NA	MP	[35]
**23**	8	0	*B. subtilis*	NA	MP	[35]
**24**	8	0	*B. subtilis*	NA	MP	[35]
**25**	0	8	*B. cinerea*	NA	RI	[35]
**21**	0	8	*A. alternate*, *A. flavus*, *F. moniliforme*, *H. tetramera* and *V. alboatrum*	NA	RI	[35]
**22**	0	8	*A. flavus*	NA	RI	[35]
**23**	0	8	*A. flavus*, *F. moniliforme*, *R. stolonifera* and *V. alboatrum*	NA	RI	[35]
**24**	0	8	*A. flavus*, *F. moniliforme* and *H. tetramera*	NA	RI	[35]
**25**	2	1	*P. phaseolicola*	NA	26 ± 0.2	[36]
**26**	2	1	*P. phaseolicola*	NA	22 ± 0.2	[36]
**27**	2	1	*P. phaseolicola*	NA	27 ± 0.2	[36]
**28**	2	1	*P. phaseolicola*	NA	23 ± 0.3	[36]
**29**	2	1	*P. phaseolicola*	NA	20 ± 0.1	[36]
**30**	2	1	*P. phaseolicola*	NA	32 ± 0.3	[36]
**31**	2	1	*P. phaseolicola*	NA	30 ± 0.1	[36]
**32**	2	1	*S. aureus*	NA	37 ± 0.1	[36]
**33**	2	1	*P. phaseolicola*	NA	32 ± 0.2	[36]
**34**	2	1	*P. phaseolicola*	NA	39 ± 0.1	[36]
**35**	6	3	*Klebsiella* and *Salmonella*	200 μg/mL	11	[37]
**36**	6	3	*Klebsiella* and *A. niger*	200 μg/mL	13	[37]
**37**	6	3	*S. aureus*	200 μg/mL	14	[37]
**38**	6	3	*S. aureus*	200 μg/mL	13	[37]
**39**	6	3	*A. niger*	200 μg/mL	14	[37]
**40**	6	3	*A. niger*	200 μg/mL	15	[37]
**41**	4	4	*P. aeruginosa*, *A. niger* and *Cladosporium*	12.5 μg/mL	MIC	[38]
**42**	4	4	*E. coli*, *S. aureus* and *A. flavus*	12.5 μg/mL	MIC	[38]
**43**	4	4	*B. subtilis*, *A. niger* and *C. albicans*	12.5 μg/mL	MIC	[38]
**44**	4	4	*E. coli*, *P. aeruginosa*, *A. niger* and *Cladosporium*	12.5 μg/mL	MIC	[38]
**45**	4	4	*S. aureus* and *A. flavus*	12.5 μg/mL	MIC	[38]
**46**	4	4	*S. aureus*, *P. aeruginosa*, *A. niger*, *A. flavus* and *C. albicans*	12.5 μg/mL	MIC	[38]
**47**	5	2	*S. typhi*	100 μg/mL	68.13	[39]
**48**	5	2	*S. typhi*	100 μg/mL	72.00	[39]
**49**	5	2	*S. typhi*	100 μg/mL	79.36	[39]
**50**	5	2	*S. typhi*	100 μg/mL	76.44	[39]
**51**	5	2	*S. typhi*	100 μg/mL	82.05	[39]
**52**	5	2	*S. typhi*	100 μg/mL	65.00	[39]
**53**	5	2	*C. albicans*	100 μg/mL	71.32	[39]
**54**	5	2	*S. typhi*	100 μg/mL	75.66	[39]
**55**	5	2	*S. typhi*	100 μg/mL	72.22	[39]
**56**	5	2	*S. typhi*	100 μg/mL	80.00	[39]
**57**	3	0	*P. aeruginosa*	200 μg/mL	21	[40]
**58**	3	0	*P. aeruginosa*	200 μg/mL	21	[40]
**59**	3	0	*P. aeruginosa*	200 μg/mL	22	[40]
**60**	3	0	*P. aeruginosa*	200 μg/mL	23	[40]
**61**	3	0	*E. coli*	200 μg/mL	24	[40]
**62**	3	0	*P. aeruginosa*	200 μg/mL	23	[40]
**63**	2	2	*E. coli*	100 μg/mL	26	[41]
**64**	2	2	*E. coli*	100 μg/mL	27	[41]
**65**	2	2	*S. aureus*	100 μg/mL	21	[41]
**66**	2	2	*S. aureus*	100 μg/mL	25	[41]
**67**	2	2	*E. coli*	100 μg/mL	22	[41]
**68**	2	2	*S. aureus*	100 μg/mL	28	[41]
**69**	2	2	*S. aureus*	100 μg/mL	23	[41]
**70**	2	2	*E. coli*, *S. aureus*	100 μg/mL	28	[41]
**71**	4	3	*E. coli*, *A. flavus*	500 μg/mL	24	[42]
**72**	4	3	*Cladosporium*	500 μg/mL	28	[42]
**73**	4	3	*A. niger*	500 μg/mL	27	[42]
**74**	4	3	*A. flavus*	500 μg/mL	26	[42]
**75**	4	3	*A. flavus*	500 μg/mL	28	[42]
**76**	4	3	*A. flavus*	500 μg/mL	29	[42]
**77**	4	3	*P. aeroginosa* *A. flavus* *A. niger*	10 10 10	80% 81% 82%	[43]
**78**	4	3	*A. niger*	10	80%	[43]
**79**	4	3	*Cladosporium*	10	90%	[43]
**80**	4	3	*Cladosporium*	10	82%	[43]
**81**	4	3	*Cladosporium*	>100	>93%	[43]
**82**	4	3	*Cladosporium*	>100	>96%	[43]
**Gentamycin**	4	0	All four	100	100%	[43]
**Fluconozole**	3	0	All three	100	100%	[43]
**83**	2	2	*P. aeroginosa*	NA	17	[44]
**84**	2	2	*P. aeroginosa*	NA	18	[44]
**85**	2	2	*E. coli*	NA	20	[44]
**86**	2	2	*P. aeroginosa*	NA	13	[44]
**87**	2	2	*P. aeroginosa*	NA	16	[44]
**88**	2	2	*P. aeroginosa*	NA	17	[44]
**89**	2	2	*P. aeroginosa*	NA	22	[44]
**90**	2	2	*P. aeroginosa*	NA	16	[44]
**91**	2	2	*E. coli* *P. aeruginosa* and *C. albicans*	NA	15	[44]
**92**	2	2	*A.niger* *C.albicans*	NA	18	[44]
**93**	2	2	*P. aeroginosa*	NA	24	[44]
**94**	2	2	*E. coli*	NA	17	[44]
**95**	2	2	*E. coli*	NA	18	[44]
**96**	2	2	*E. coli* *C. albicans*	NA	17	[44]
**Fluconozole**	0	2	*A. niger*	NA	24	[44]
**Ciprofloxacin**	2	0	*P. aeroginosa*	NA	30	[44]
**97**	2	2	*S. aureus*	3.12	21	[45]
**98**	2	2	*S. aureus*		19	[45]
**99**	2	2	*S. aureus*	1.56	22	[45]
**100**	2	2	*S. aureus* *E. coli*		18	[45]
**101**	2	2	*S. aureus* *E. coli*	3.12	21	[45]
**102**	2	2	*S. aureus*		20	[45]
**103**	2	2	*S. aureus*	1.56	25	[45]
**104**	2	2	*S. aureus*		20	[45]
**Norfloxacin**	2	0	*S. aureus* *E. coli*	1.56	26	[45]
**Griseofulvin**	0	2	*A. niger* and *C. albicans*	1.56	26	[45]
**105**	4	3	*Cladosporium*	10	>59%	[46]
**106**	4	3	*Cladosporium*	10	>55%	[46]
**107**	4	3	*Cladosporium*	10	>65%	[46]
**108**	4	3	*Cladosporium*	10	>61%	[46]
**109**	4	3	*E. coli* and *A. flavus*	25	>20% >16%	[46]
**110**	4	3	*E. coli*	25		[46]
**Gentamycin**	4	0	*E. coli*	25	86%	[46]
**Flucanozole**	0	3	*Cladosporium*	25	92%	[46]
**111**	4	3	*A. flavus*	25	80%	[47]
**112**	4	3	*A. flavus*	25	77%	[47]
**113**	4	3	*A. niger* *A. flavus*	25 25	83% 71%	[47]
**114**	4	3	*A. niger* and *S. aureus*	10		[47]
**115**	4	3	*A. niger*	25	79%	[47]
**116**	4	3	*A. flavus*	25	72%	[47]
**117**	4	3	*A. niger* and *P. aeruginosa*	25 25	82% 66%	[47]
**118**	4	3	*A. niger*	25	70%	[47]
**119**	4	3	*A. flavus*	25	78%	[47]
**120**	4	3	*A. flavus*	10		[47]
**121**	4	3	*A. flavus* *A. niger*	25 25	80% 74%	[47]
**122**	4	3	*A. flavus*	25	85%	[47]
**Gentamycin**	4	3	All four	25	100%	[47]
**Flucanozole**	4	3	All three	25	100%	[47]
**123**	4	0	*B. subtili*, *E. coli* and *P. aeruginosa*	NA	13 mm	[48]
**124**	4	0	*B. subtili* *S. aureus* and *P. aeruginosa*	NA	18 mm	[48]
**125**	4	0	*B. subtili* and *P. aeruginosa*	NA	17 mm	[48]
**126**	4	0	*E. coli*	NA	17 mm	[48]
**127**	4	0	*S. aureus* and *E. coli*	NA	18 mm	[48]
**128**	4	0	*B. subtili*	NA	18 mm	[48]
**129**	4	3	*F. oxysporum* and *S. aureus*	100 ppm 500 ppm	79 ± 1.4% 15 ± 0.03%	[49]
**130**	4	3	*F. oxysporum* *S. aureus*	100 ppm 500 ppm	71 ± 0.4% 13.9 ± 0.09%	[49]
**131**	4	3	*F. oxysporum* and *S. aureus*	100 ppm 500 ppm	84 ± 0.6% 14.4 ± 0.1%	[49]
**132**	4	3	*C. albicans* and *S. aureus*	100 ppm 500 ppm	59 ± 0.7% 12.6 ± 0.09%	[49]
**133**	4	3	*C. albicans* and *P. aeruginosa*	100 ppm 500 ppm	60 ± 0.4% 12.0 ± 0.03%	[49]
**134**	4	3	*F. oxysporum* and *E. coli*	100 ppm 500 ppm	69 ± 0.2% 10.5 ± 0.09%	[49]
**Ciprofloxacin**	4	0	*S. aureus*	500 ppm	18.6 ± 0.03%	[49]
**Fluconazole**	0	3	*F. oxysporum*, *C. albicans* and *A. Niger*	500 ppm	100	[49]
**135**	1	0	*F. psychrophilum*	32	16.1 ± 0.9%	[50]
**136**	4	1	*A. Niger*	NA	4.6 mm	[51]
**137**	4	1	*A. Niger*	NA	1.15 mm	[51]
**138**	4	1	*S. aureus*	NA	0.81 mm	[51]
**139**	4	1	*S. aureus*	NA	1.72 mm	[51]
**140**	4	1	*S. aureus*	NA	1.72 mm	[51]
**141**	4	2	*R. oryzae*	200	10 mm	[11]
**142**	4	2	*A. niger*	200	11 mm	[11]
**143**	4	2	*P. auregenosa* *A. niger* *R. oryzae*	200 200 200	12 mm 12 mm 12 mm	[52]
**144**	4	2	*A. niger*	200	11mm	[52]
**145**	4	2	*A. niger*	200	14 mm	[52]
**146**	4	2	*A. niger*	200	15 mm	[52]
**Gentamicin**	2	0	*P. auregenosa*	200	15 mm	[52]
**Fluconazole**	0	2	*A. niger*	200	16 mm	[52]
**147**	4	0	*K. pneumonia*	100	22 mm	[53]
**148**	0	2	*O. latemarginatus*	100	82.2 ± 1.1	[54]
**149**	0	2	*O. latemarginatus*	100	74.4 ± 1.1	[54]
**150**	0	2	*O. latemarginatus*	100	83.7 ± 0.6	[54]
**151**	0	2	*O. latemarginatus*	100	76.7 ± 1.1	[54]
**152**	0	2	*O. latemarginatus*	100	67.8 ± 1.1	[54]
**153**	1	1	*E. coli* and *A.* *niger*	20 µg/mL	MIC	[55]
**154**	1	1	*E. coli* and *A.* *niger*	20 µg/mL	MIC	[55]
**155**	1	1	*E. coli and A. niger*	20 µg/mL	MIC	[55]
**156**	1	1	*E. coli and A. niger*	20 µg/mL	MIC	[55]
**157**	0	1	*Candida*	5.2 µM	MIC	[56]
**158**	0	1	*Candida*	10.4 µM	MIC	[56]
**159**	0	1	*Candida*	16.7 µM	MIC	[56]
**160**	0	1	*Candida*	8.2 µM	MIC	[56]
**161**	0	1	*Candida*	NA	MIC	[56]
**162**	0	1	*Candida*	14.5 µM	MIC	[56]
**163**	0	1	*Candida*	3.6 µM	MIC	[56]
**164**	0	1	*Candida*	4.4 µM	MIC	[56]
**165**	0	1	*Candida*	0.7 µM	MIC	[56]
**166**	0	1	*Candida*	9.8 µM	MIC	[56]
**167**	0	1	*Candida*	12.6 µM	MIC	[56]
**Amphotericin B**		1	*Candida*	0.7 µM	MIC	[56]
**Ketoconazole**		1	*Candida*	4.7 µM	MIC	[56]
**168**	3	0	*K. pneumoniae* and *S. aureus*	100 µg/mL	19 ± 0	[57]
**169**	3	0	*K. pneumoniae* and *S. aureus*	100 µg/mL	18 ± 0	[57]
**170**	3	0	*K. pneumoniae*, and *S. aureus*	100 µg/mL	17 ± 0	[57]
**171**	3	0	*K. pneumoniae*, *E. coli*, and *S. aureus*	100 µg/mL	16 ± 0	[57]

MIC: Minimum inhibitory concentration; MP: Mortality percentage; RI: Radial inhibition; NA: Not applicable.

**Table 3:** Antioxidant activity of selected coumarin-derived imine–metal complexes.

Compound	Scavenging Activity (%)	Ref.
**41**	44	[38]
**42**	59	[38]
**43**	48	[38]
**44**	63	[38]
**45**	58	[38]
**46**	61	[38]
**BHA**	70	[38]

**Table 4:** Anthelmintic activity of the coumarin-derived imine–metal complexes.

Compound	Conc.(μg/mL)	Time of Paralysis (min)	Time of Death (min)	Ref.
**Albendazole**	10	3.48 ± 0.06	7.25 ± 0.14	[33]
**13**	10	7.40 ± 0.04	10.12 ± 0.03	[33]
**14**	10	9.13 ± 0.01	15.51 ± 0.00	[33]
**15**	10	5.39 ± 0.22	9.31 ± 0.01	[33]
**13**	2	12.14 ± 0.14	19.50 ± 0.02	[33]
**14**	2	16.10 ± 0.25	21.20 ± 0.09	[33]
**15**	2	10.27 ± 0.03	16.41 ± 0.06	[33]
**35**	10	4.30 ± 0.04	7.72 ± 0.03	[37]
**36**	10	3.43 ± 0.01	6.51 ± 0.20	[37]
**37**	10	3.29 ± 0.02	6.91 ± 0.01	[37]
**38**	10	54 ± 0.01	7.25 ± 0.01	[37]
**39**	10	3.25 ± 0.00	6.93 ± 0.10	[37]
**40**	10	3.20 ± 0.04	6.25 ± 0.12	[37]
**35**	2	8.24 ± 0.04	16.50 ± 0.09	[37]
**36**	2	7.20 ± 0.05	120.60 ± 0.09	[37]
**37**	2	7.17 ± 0.01	19.61 ± 0.06	[37]
**38**	2	7.10 ± 0.023	11.41 ± 0.05	[37]
**39**	2	6.10 ± 0.01	12.15 ± 0.09	[37]
**40**	2	5.14 ± 0.03	10.20 ± 0.09	[37]

## Data Availability

Not applicable.
